# Reactive Oxygen Species in Macrophages: Sources and Targets

**DOI:** 10.3389/fimmu.2021.734229

**Published:** 2021-09-30

**Authors:** Marcella Canton, Ricardo Sánchez-Rodríguez, Iolanda Spera, Francisca C. Venegas, Maria Favia, Antonella Viola, Alessandra Castegna

**Affiliations:** ^1^ Department of Biomedical Sciences, University of Padova, Padova, Italy; ^2^ Fondazione Istituto di Ricerca Pediatrica Città della Speranza - IRP, Padova, Italy; ^3^ Department of Biosciences, Biotechnologies and Biopharmaceutics, University of Bari, Bari, Italy

**Keywords:** macrophages, reactive oxygen species (ROS), mitochondria, innate immunity, redox signaling, inflammasome, monoamine oxidase, protein oxidation

## Abstract

Reactive oxygen species (ROS) are fundamental for macrophages to eliminate invasive microorganisms. However, as observed in nonphagocytic cells, ROS play essential roles in processes that are different from pathogen killing, as signal transduction, differentiation, and gene expression. The different outcomes of these events are likely to depend on the specific subcellular site of ROS formation, as well as the duration and extent of ROS production. While excessive accumulation of ROS has long been appreciated for its detrimental effects, there is now a deeper understanding of their roles as signaling molecules. This could explain the failure of the “all or none” pharmacologic approach with global antioxidants to treat several diseases. NADPH oxidase is the first source of ROS that has been identified in macrophages. However, growing evidence highlights mitochondria as a crucial site of ROS formation in these cells, mainly due to electron leakage of the respiratory chain or to enzymes, such as monoamine oxidases. Their role in redox signaling, together with their exact site of formation is only partially elucidated. Hence, it is essential to identify the specific intracellular sources of ROS and how they influence cellular processes in both physiological and pathological conditions to develop therapies targeting oxidative signaling networks. In this review, we will focus on the different sites of ROS formation in macrophages and how they impact on metabolic processes and inflammatory signaling, highlighting the role of mitochondrial as compared to non-mitochondrial ROS sources.

## 1 Introduction

The immune system orchestrates a complex defensive strategy against pathogens or tissue injury. In vertebrates, two types of immunity are used to protect the host from infections: innate and adaptive. The innate system, which constitutes the first line of defense, is genetically programmed to recognize structures that are broadly shared by invading microbes (named PAMPs, pathogen-associated molecular patterns) and by cell damage (named DAMPs, damage-associated molecular patterns). Cells of the innate immune system include macrophages, dendritic cells, neutrophils, eosinophils, basophils, mast cells and Natural Killer cells. In contrast, the adaptive system, also referred as the acquired immune system, employs antigen-specific receptors that are specifically developed (“acquired”) by lymphocytes during the lifetime of the organism.

Macrophages are large, specialized cells that rapidly recognize, engulf, and destroy pathogens or apoptotic cells. Indeed, the term macrophage is formed by the combination of the Greek terms “makro” meaning big and “phagein” meaning eat. One of the fundamental features of macrophages is their high plasticity, which allows them to respond to stimuli from the complex tissue microenvironment, by changing rapidly their functional profile through a process named “polarization”. In fact, they initially adopt a proinflammatory phenotype and then later they acquire an anti-inflammatory profile to repair the tissue damage (1, 2). Due to the complex stimulating network, the process of macrophage polarization in an *in vivo* setting cannot be recapitulated by the static vision of M1-M2 polarization adopted in *in vitro* experiments, reached by stimulation with lipopolysaccharide (LPS)/interferon-γ (IFN-γ) or Interleukin (IL)-4/IL-13 or IL-10, respectively (2, 3). However, macrophages with predominantly proinflammatory properties are commonly referred as M1, whereas those with a pro-fibrotic and anti-inflammatory signature as M2. They are found ubiquitously in tissues as resident cells patrolling their surroundings, thus maintaining tissue integrity. Resident macrophages have different names according to where they function in the body. For instance, macrophages in brain are termed microglia, while in liver they are called Kupffer cells (4). Moreover, in case of tissue damage or infection, monocytes leave the bloodstream to enter the affected tissues and undergo a series of changes to become macrophages.

Reactive oxygen species (ROS) have been known for many years as fundamental for macrophages to kill invasive microorganisms through the oxidative burst mediated by NADPH oxidase (5). However, more recent studies have shown that mitochondrial ROS play essential roles in several innate immune functions, through subtle changes in the intracellular redox state (6).

Oxygen is a highly electronegative element that readily accepts electrons generated by normal oxidative metabolism within cells, thereby producing ROS. The term “ROS” includes superoxide anion (O_2_ **^-.^**), hydrogen peroxide (H_2_O_2_), hydroxyl radical and singlet oxygen, which are produced as described in (6). Besides ROS, other endogenous small, reactive signaling molecules include reactive nitrogen species (RNS), such as nitric oxide, as well as hydrogen sulfide and carbon monoxide. Several reviews discuss their roles in macrophage function (7–9), so they will not be considered in the present review.

While ROS have been considered for a long time as dangerous by-products of mitochondrial metabolism, it is now widely accepted that they play crucial roles as signaling molecules, regulating cell growth, differentiation, and apoptosis (10). As discussed extensively in other reviews (5, 6), ROS appear to play different -sometime opposing- roles depending on their subcellular origin and levels. Among ROS, H_2_O_2_ is by far the most prevalent and best studied cellular oxidant and plays a major role in redox regulation of biological activities (11). Similar to other signaling molecules, the intracellular concentrations of H_2_O_2_ are maintained very low (in the range of 1-100 nM) and are tightly regulated (6). They are produced in such a low level to be confined to a restricted subcellular location and to induce signaling pathways, supporting normal physiological processes. This range of concentration must be considered just as an order of magnitude, because it depends on many factors, as cell type, local concentrations, etc. Different stimuli, such as growth factors or chemokines, trigger a physiological increase of H_2_O_2,_ which targets specific proteins leading to their reversible oxidation, thereby altering their activity, localization and interactions. These protein modifications contribute to orchestration of various processes in cells and organs, including cell proliferation, differentiation, migration and angiogenesis. On the other hand, high levels of ROS (roughly reaching micromolar concentrations) are likely to become involved in non-specific oxidation of targets, causing damage to macromolecules, impairing their function and triggering stress response mechanisms, as inflammation, fibrogenesis, tumor growth, metastasis and, at higher levels, cell death (6, 12). In this review, we will focus on the different sites of ROS formation in macrophages, the major molecular redox targets, and their related cellular response.

## 2 Sources of ROS in Macrophages

### 2.1 Cytosolic Sources

#### 2.1.1 NADPH Oxidases

**Figure 1:**
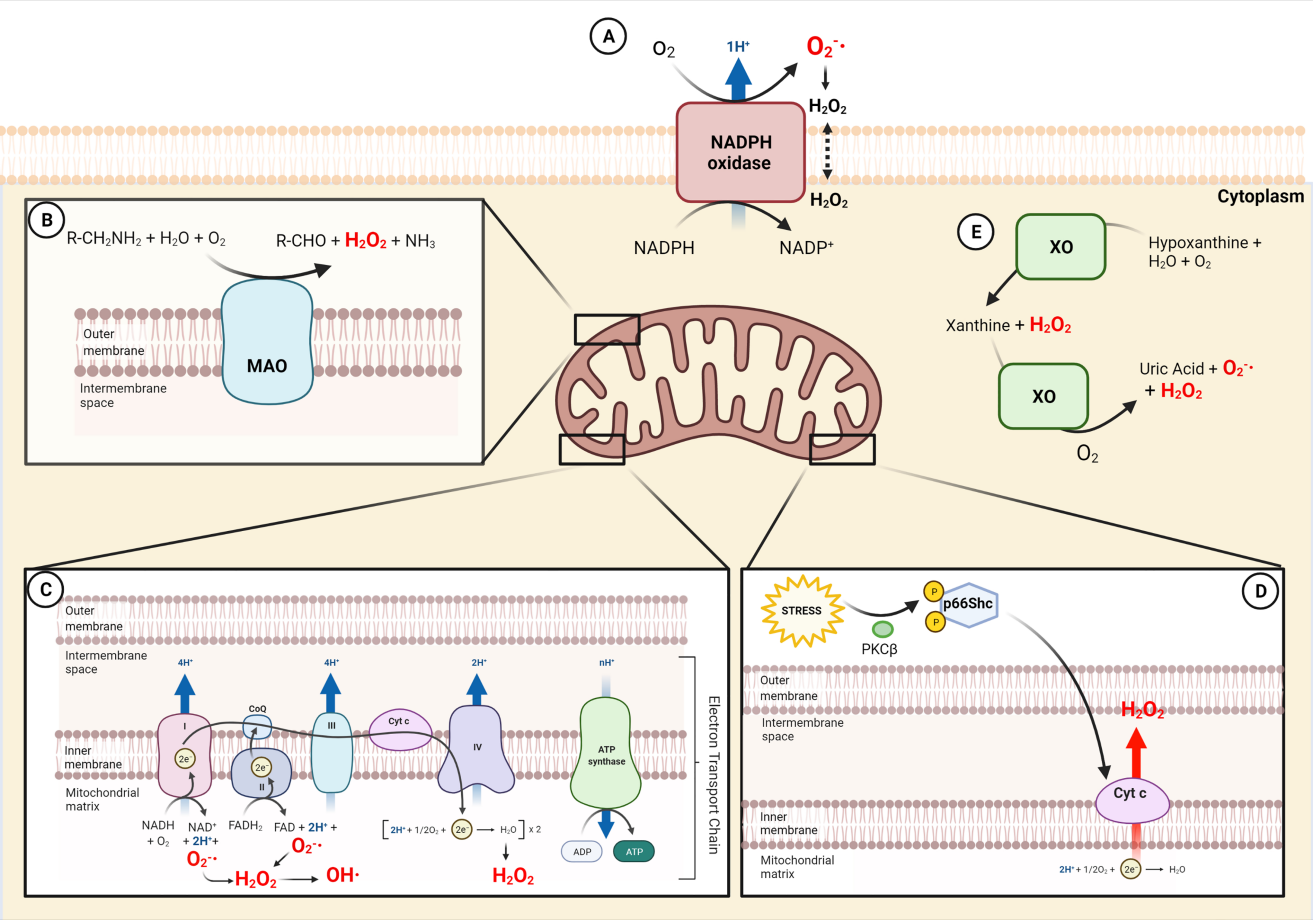
Sources of ROS in macrophages. **(A)** Generation of $O_{2}^{-\cdot }$ and consecutive formation of H_2_O_2_ by NADPH oxidase (NOX) in the external cell membrane. **(B)** Production of H_2_O_2_ in the outer mitochondria membrane by oxidative deamination of biogenic and xenobiotic amines by MAO. **(C)** Electron Transport chain in the inner mitochondrial membrane generates $O_{2}^{-\cdot }$ , H_2_O_2_ and OH· in the mitochondrial matrix. **(D)** Cytochrome *c* in the inner mitochondrial membrane produces H_2_O_2_ following p66Shc activation by stress. **(E)** The xanthine metabolism produces H_2_O_2_ and $O_{2}^{-\cdot }$ by XO in the cytoplasm. NADP^+^, Nicotinamide adenine dinucleotide phosphate; MAO, monoamine oxidases; CoQ, coenzyme Q; FAD, flavin adenine dinucleotide; Cyt *c*, cytochrome *c*; ADP, adenosine diphosphate; ATP, adenosine triphosphate; PKCβ, protein kinase C β; XO, xanthine oxidase.

NADPH oxidases (NOXs) are a family of transmembrane enzymes specifically dedicated to produce cytosolic ROS (cytROS) (13, 14), mainly located in the plasma membrane (**Figure 1**). NOXs catalyze superoxide formation by transferring one electron from NADPH to oxygen (15). Superoxide can be further converted to H_2_O_2_ either by spontaneous dismutation or catalyzed by superoxide dismutase. So far, seven members of the family have been described (NOX1-5 and Duox1-2) (16), and three of them (NOX1, NOX2 and NOX4) have been identified in phagocytes (17–27). NOX2 is the most well-characterized isoform for its role in phagocytic function. During phagocytosis, the plasma membrane is internalized and becomes the interior wall of the phagocytic vesicle. Next, the $O_{2}^{-\cdot }$ produced by NOX2 (named “oxidative burst”) is released into the vesicle to kill the internalized target. The relevance of cytROS in host immunity has been demonstrated in chronic granulomatous disease (CGD), a genetic disorder characterized by mutations in genes encoding components of the NOX2 complex (28, 29). Patients with CGD are hypersensitive to both bacterial and fungal infections, as their phagocytic cells fail in killing pathogens due to the extremely low oxidative burst during phagocytosis (30, 31). More recently, it has been shown that also NOX4-mediated ROS production is selectively required for the host defense against *Toxoplasma gondii* infection (26).

Besides killing invasive microorganisms, NOX-dependent ROS production influences many metabolic processes and disease states. For instance, NOX1 and NOX2 are critical for the differentiation of monocytes to macrophages and the M2-type polarization, as assessed in macrophages from NOX1/NOX2 double knockout mice (19). Interestingly, ROS generated by NOX2 were found to contribute to fatty liver disease (27). Indeed, *Nox2*-deficient mice were protected against hepatic steatosis induced by high-fat diet and insulin resistance (27). Mechanistically, palmitate triggers endocytosis of the Toll-like receptor 4 (TLR4)-MD2 complex, leading to NOX2 activation, ROS generation and proinflammatory cytokine production in hepatic infiltrating macrophages (27). By the way, although palmitate has been considered a TLR4 ligand, a subsequent study demonstrated that it does not directly bind this receptor (32). More recently, NOX1-dependent ROS production has been found to be neurotoxic for microglia located in the retina (33). Its overactivation is mediated by the translocator protein TSPO. Using different NOX-deficient mice, the study shows that the TSPO-NOX1 axis controls the phagocyte-triggered angiogenesis in the eye of a mouse model of age-related macular degeneration, a major cause of blindness in the elderly (33). NOX4 has also been shown to be an inducible source of ROS, driving cell death when monocytes and macrophages were exposed to oxidized low density lipoproteins (oxLDL) (17).

Finally, different studies investigated the impact of NOX in the activation of the NLRP3 inflammasome in macrophages. The NLRP3 inflammasome is a molecular platform activated upon signs of cellular danger (PAMP or DAMP) to trigger innate immune defenses through the maturation of pro-inflammatory cytokines (34, 35). For its activation it requires the adapter protein apoptosis associated speck-like protein containing a CARD (ASC) to activate caspase-1, which cleaves pro-interleukin (IL)-1β and pro-IL-18 in IL-1β and IL-18 (34). The results linking NOX to the NLRP3 inflammasome are controversial, and this could be ascribed to species differences, differential regulation of monocytes and macrophages or redundant NOX enzymes, as discussed (23, 36, 37). Also the localization of NOX4 is quite arguable/disputed, as some studies found this isoform even in mitochondria, others in the plasma membrane and in the endoplasmic reticulum (6).

#### 2.1.2 Xantine Oxidase

Xanthine dehydrogenase is an enzyme that can be converted to its oxygenase form xantine oxidase (XO) upon oxidative stress. Both forms generate uric acid from hypoxanthine or xanthine, but XO also produces ROS (38) (**Figure 1**). The conversion of xanthine dehydrogenase to XO can be due to irreversible proteolytic cleavage, or to reversible sulfhydryl modification (39, 40). Its role has been widely studied for many years, but scarce information is available with respect to the innate immune system. Interestingly, convincing evidence reports that XO is a source of ROS that mediates NLRP3 inflammasome activation in macrophages (41) and that represents a key factor to trigger inflammation against parasitic infection (42).

### 2.2 Mitochondrial Sources

Mitochondria are important sources of ROS, as they are the main oxygen consumers in the cell (43). These organelles produce ROS through various mechanisms, including electron leak from the electron transport chain to oxygen, or as by-products of the catalytic activity of several oxidases (44–46). Interestingly, it has been suggested that also mitochondrial ROS (mtROS) represent an important component of the antibacterial responses, thereby revealing a novel pathway linking innate immune signaling to mitochondria (47). Specifically, the engagement of a subset of Toll-like receptors (TLR1, TLR2 and TLR4) was found to cause the recruitment of mitochondria to macrophage phagosomes and augments mtROS, although the mechanism is still partially unclear (47). Another study highlighted the relevance of mtROS as compared to cytROS. Bulua and Coworkers showed that mtROS are responsible for excessive LPS-driven production of proinflammatory cytokines in cells from patients with an autoinflammatory disorder caused by missense mutations in the type-1 TNF receptor (TNFR1), named TNF receptor-associated periodic syndrome (TRAPS) (48). On the other hand, NOXs are not the source of proinflammatory ROS, as NOX subunits were found to be dispensable for inflammatory cytokine production (48). The authors hypothesized several mechanisms by which TNFR1 mutations enhance mitochondrial respiration, although further research should more specifically address this issue. The endoplasmic reticulum, where mutant TNFR1 resides, can provide signals to activate mitochondrial respiration (49). Moreover, mutant TNFR1 may increase activation of the riboflavin kinase, which can associate with TNFR1 (50), possibly leading to enhanced charging of FAD-dependent enzymes in the mitochondria.

#### 2.2.1 Electron Transport Chain

The electron transport chain (ETC) is a series of electron-carrier proteins located in the mitochondrial inner membrane (**Figure 1**). It transfers electrons from the reduced coenzymes NADH and FADH_2_, generated by catabolic processes, to molecular oxygen. Thanks to this process, three of the four protein complexes pump protons across the mitochondrial inner membrane to maintain the protonmotive force driving ATP synthesis. A physiological consequence of the electron transfer is the generation of mitochondrial ROS (mtROS). In fact, the electron leak from complexes I, II, and III mediates the one-electron reduction of oxygen to superoxide ( $O_{2}^{•-}$ ), which can then be rapidly converted to H_2_O_2_ by manganese superoxide dismutase (MnSOD) within the mitochondrial matrix. H_2_O_2_ can then freely diffuse in the cell and trigger thiol oxidation of proteins.

mtROS can be also produced at complex I through *reverse electron transfer (RET)*. This process, observed *in vitro* in the sixties of the last century (51, 52), has been considered of uncertain physiological relevance for many years. More recently, several studies highlighted that RET at complex I is a process underlying mitochondrial redox signaling in physiological and pathological conditions (45, 53–56). It occurs when electrons flow back through complex I, in contrast to conventional forward transport, because of elevated mitochondrial membrane potential coupled to highly reduced coenzyme Q. Thus, the electrons can reduce NAD^+^ to NADH and drive superoxide formation. Indeed, in macrophages stimulated with LPS, Mills et al. showed that the accumulation of succinate, that is oxidized by complex II (succinate dehydrogenase), results in mtROS production, seemingly from RET at complex I (45).

#### 2.2.2 Monoamine Oxidases

A relevant mitochondrial-specific source of ROS is monoamine oxidase (MAO), although its impact on inflammation has been quite overlooked. MAO is located in the outer mitochondrial membrane and catalyzes the oxidative deamination of neurotransmitters (i.e. catecholamines) and dietary amines, generating aldehydes, ammonia and H_2_O_2_ (57) (**Figure 1**). The two isoforms MAO-A and MAO-B differ for substrate specificity and inhibitor sensitivity. MAO-A has greater affinity for hydroxylated amines, i.e. serotonin and noradrenaline, whereas MAO-B has greater affinity for non-hydroxylated ones, i.e. β-phenylethylamine. Notwithstanding, they show similar affinity for dopamine and tyramine. MAO physiologic role is well established in the central nervous system: it terminates neurotransmitter signaling and, by doing this, it generates H_2_O_2_ that is constantly removed by endogenous scavengers (57). On the contrary, in pathological conditions, the increased activity of the enzyme overcomes the cellular antioxidant defenses, altering the redox homeostasis and eliciting deleterious effects, as in muscular dystrophy and cardiac injury (58–62). Only few studies have characterized the role of MAO in innate immunity. It has been shown that MAO-A is upregulated by LPS or by IL-4/IL-13 in phagocytic cells (63–66). These studies also suggest that IL-13 and IL-4 induced MAO-A-mediated ROS generation through Jak signaling pathways (65).

Few years ago, a study from Tschopp’s group highlighted the crucial role of mtROS in NLRP3 inflammasome activation, although the source of ROS production was unclear (65, 67). Recently, it has been shown that H_2_O_2_ produced by MAO-B plays a non-redundant role in sustaining NLRP3 inflammasome activation (68) in human and murine macrophages. Mechanistically, MAO-B-dependent ROS formation causes mitochondrial dysfunction and NF-κB activation, resulting in NLRP3 and pro-IL-1β overexpression. Both *in vitro* and *in vivo*, MAO-B inhibition by the clinical grade drug rasagiline prevents IL-1β secretion, and MAO-B deficient mice display an impaired response to LPS-mediated endotoxemia (68).

Remarkably, two interesting studies highlight the critical role of MAO in macrophages, considering the enzyme as a catecholamine consumer and not as a ROS producer. Briefly, macrophages from adipose tissue of aged mice displayed upregulation of MAO-A in a NLRP3 inflammasome-dependent manner. The enhanced activity of MAO-A increases catecholamine catabolism, thereby dampening the activation of lipolytic signaling in adipocytes as this process depends on noradrenaline levels (69). In the same context, MAO-A was identified in a subset of cells called macrophages associated with sympathetic neurons (SAMs) that work in the clearance of noradrenaline, acting as a sink (70). Moreover, SAMs were increased in two models of obesity and contributed to the disease by excessive import and metabolism of noradrenaline (70, 71).

#### 2.2.3 P66shc

p66Shc is a cytosolic adaptor protein that regulates the cellular redox state and apoptosis (72, 73). Oxidative stress activates protein kinase C-β (PKCβ), which phosphorylates p66Shc. As a result, p66Shc translocates to mitochondria, where it generates H_2_O_2_. Indeed, this redox enzyme utilizes reducing equivalents of the mitochondrial ETC through the oxidation of cytochrome *c* to catalyze the partial reduction of molecular oxygen (72). Mice lacking p66Shc are long-lived, and their cells are both resistant to oxidative stress and produce less ROS. A few studies characterized p66Shc in the immune system and were mainly focused on its role in the macrophages of the atherosclerotic lesions induced by high-fat diet or diabetes (74, 75). Chronic high-fat diet was reported to increase the atherosclerotic lesion area more in wild-type than *p66Shc* knockout mice. Early lesions from *p66Shc* knockout mice had fewer macrophage-derived foam cells as compared to those from wild-type mice (74). A cross-talk between p66Shc and NOX has been observed in murine macrophages, as NOX activation is defective in p66Shc-deficient mice, leading to decreased superoxide production (76).

## 3 Targets of ROS in Macrophages

It is widely accepted that physiological levels of ROS can cause reversible post-translational modifications in proteins to regulate signaling pathways. More in detail, H_2_O_2_ can oxidize thiol groups (-SH) on cysteine residues to form sulfenic acid (-SOH), which can react with GSH to become glutathionylated (-SSG), or with adjacent thiols to form a disulfide bond (-SS-) (6, 77). Cysteine thiols can be considered as redox sensors, as they are particularly sensitive to oxidants. The reactivity of thiols for ROS is limited to the cysteinyl residues placed in sites that enable the formation of the thiolate form (-S^-^), which is more nucleophilic and susceptible to oxidation (6). These modifications can change the activity of the target proteins, thus altering their function and their downstream signaling pathway and metabolism (78). Supraphysiological concentration of ROS leads to oxidative stress, a state of imbalance between ROS production and ROS removal, which can be either due to increased ROS formation or to reduced antioxidant defenses.

In this review, we summarize several examples of signaling targets for ROS that have been identified in macrophages, as the list of targets is too extensive to be covered exhaustively (6). Importantly, cysteine oxidation networks have been recently become accessible (79) and provide a quantitative tissue-specific overview of the redox-regulated proteome, called the Oximouse dataset (79).

### 3.1 Nrf2/Keap1 Complex

Nrf2 (nuclear factor-erythroid 2 p45-related factor 2) is the master regulator of the antioxidant response: it is responsible for maintaining the redox homeostasis under oxidative stress by regulating the expression of detoxifying enzymes involved in glutathione, NADPH and thioredoxin systems (including GCLC, GCLM, NQO1, G6DH, TRX and HO-1) (12, 80, 81). Under basal conditions, Nrf2 is constitutively degraded by the proteasome, as its cytosolic repressor Keap1 (Kelch-like ECH-associated protein 1) enables the Cul3-Ring-box 1 E3 ubiquitin ligase to ubiquitinate it. Oxidative stress induces the oxidation of Cys151, Cys273 and Cys288 in two domains of the negative regulator Keap1, inducing its conformational change and disruption of the Nrf2/Keap1 interaction (82). This results in the stabilization of Nrf2, which translocates to the nucleus and binds specific DNA sequences, the Antioxidants Response Elements (ARE) (83–85). The role of Nrf2 in macrophages is still controversial, as both anti-inflammatory and pro-inflammatory mechanisms have been described, as summarized here below. Several studies demonstrate that the Keap1/Nrf2/ARE signaling pathway attenuates inflammation. Nfr2 deletion causes exacerbation of inflammation in different experimental murine models, such as septic shock and emphysema (86–88), and antioxidant treatments reduce these effects. A recent study reports that murine macrophages achieve self-protection against oxidative stress through the Mst-Nrf2 axis (89). The kinases Mst1/2 sense ROS and maintain the cellular redox balance by modulating Nrf2 stability. Mechanistically, both phagosomal and mtROS activate Mst1/2, which phosphorylate Keap1, thereby blocking Nrf2 ubiquitination and degradation to protect cells against oxidative damage and to maintain the phagocytosis properties (89). Nrf2 was found to inhibit the NLRP3 inflammasome assembly by buffering ROS and controlling the expression of thioredoxin, which inhibits a protein necessary for NLRP3 complex stabilization, the thioredoxin-interacting protein (TXNIP) (90) (see 3.6 *Thioredoxins and ASK1* for further details). On the other hand, other studies provide evidence for a proinflammatory role. Indeed, Nrf2 plays an essential role in ROS-mediated inflammasome activation, as Nrf2-deficient macrophages show a reduced formation of ASC specks and IL-1β release in response to NLRP3 and AIM2 inflammasome stimuli (91). ASC is an adaptor protein which assembles into a large protein complex, termed “speck”, upon inflammasome activation. Hence, ASC speck formation is commonly used as a simple upstream readout for inflammasome activation (92).

The paradoxical role of Nrf2 might be related to the kind of inflammatory stimuli and/or the timing of activation. In fact, the early activation of Nrf2 triggered by antioxidant treatments can enhance the antioxidant response, reduce ROS levels and induce an anti-inflammatory signature, whereas the late activation can mainly contribute to the NLRP3 assembling, such as the one triggered by cholesterol crystals.

Despite its strong association with redox biology, the activation of Nrf2 by mtROS induces also the overexpression of macrophage-specific genes that are not classified as anti-oxidative stress-response genes, such as the gene encoding MARCO, a scavenger receptor required for bacteria phagocytosis (93). Furthermore, Nrf2 can inhibit the expression of proinflammatory cytokine genes in a redox-independent manner. Indeed, Nrf2 was found to inhibit the recruitment of RNA polymerase II onto the proinflammatory cytokine gene loci for Il-1β and Il-6 (94).

### 3.2 HIF Pathway

Hypoxia Inducible factors (HIFs) are transcription factors, consisting of an oxygen-labile subunit (HIF-α) and a constitutively stable subunit (HIF-β), which play pivotal roles in inducing cellular responses to hypoxia and in regulating immune cell effector functions (95, 96). They form a heterodimeric complex, which binds to hypoxia-responsive elements (HREs), thus activating the transcription of their target genes and promoting a metabolic and functional cell reprogramming (97). In homeostatic conditions, HIF is hydroxylated by prolyl hydroxylase (PHD), an enzyme belonging to the 2-oxoglutarate (2-OG)-dependent dioxygenase (2-OGDD) family, which catalyzes the conversion of 2-OG to succinate and employs molecular oxygen for HIF hydroxylation. The hydroxylation of HIF drives its ubiquitylation by the E3 ligase von Hippel–Lindau tumour-suppressor protein (VHL) and its consequent degradation by the proteasome (5). Upon LPS stimulation, macrophages reprogram their metabolism towards glycolysis, thereby driving ROS generation which can sustain hypoxic adaptation by HIF stabilization (3, 45, 98) and concomitantly support the expression of cytokines, such as IL-1β *via* HIF-1α. The crucial role of ROS is documented by a strong body of evidence. Limiting ROS production by uncoupling mitochondria or by expressing the alternative oxidase (AOX) inhibits the inflammatory phenotype of macrophages (45). Moreover, ROS contribute to HIF-1α stabilization by (I) diverting the PHD substrate 2-OG toward a non-enzymatic decarboxylation (99), and (II) oxidizing the PHD cofactor Fe^2+^ to Fe^3+^ (100). Finally, ROS act as inhibitor of Factor Inhibiting HIF (FIH), an asparaginyl hydroxylase belonging to the 2-OGDD family. FIH hydroxylation, occurring in a different site from PHD hydroxylation (101), impairs HIF-1α function by reducing its C-terminal transactivation domain activity (101). It is worth noting that HIF asparaginyl hydroxylation (OH) differs from the prolyl OH in the fact that is more sensitive to low concentrations of H_2_O_2_ than prolyl OH, whereas in moderate hypoxia asparaginyl OH is less effectively inhibited than prolyl OH. This suggests that hypoxia and ROS can provide different levels of regulation of HIF transcriptional output (101). In support of this finding, although in cancer cell lines, mtDNA depletion is sufficient to prevent HIF-1α stabilization under hypoxia (102). In bone-derived murine macrophages mtROS generated by spermidine activate AMP-activated protein kinase (AMPK), which in turn enhances mitochondrial function, and upregulates HIF-1α (103). Moreover, HIF-1α reduces mitochondrial mass through mitophagy, thus limiting oxygen consumption. MtROS target SDH subunit A (SDHA), leading to the inhibition of its enzymatic activity, which in turn stabilizes HIF-1α and causes the subsequent, sustained expression of IL-1β together with TCA intermediates accumulation (104).

HIF-1α mediates the expression of genes encoding for glycolytic enzymes, for the glucose transporter GLUT1 (98), and the pyruvate dehydrogenase kinase isoform 1 (PDK1) in macrophages (105, 106). When HIF1 is stabilized, PDK activity inhibits the pyruvate dehydrogenase complex, limiting the flux of pyruvate into the tricarboxylic acid (TCA) cycle. This attenuates mitochondrial respiration through ETC flux, preventing mtROS production from overpowering the antioxidant endogenous defense in hypoxic conditions (107, 108). In this scenario, PDK1 activation might also represent a strategy to divert glucose from glycolysis to the pentose phosphate pathway, leading to a higher NADPH/NADP^+^ ratio that might prevent or compensate for uncontrolled oxidative stress, which could be harmful. Indeed, LPS lethality in mice can be prevented by limiting ROS production (45). PDK activity can also be inhibited by mtROS, providing a regulatory flexibility that is functionally important in the migration setting of macrophages (109).

Besides PHD, ROS can also inhibit the activity of Jumonji domain-containing histone demethylase (JMJD), a family of Fe^2+^-dependent 2-OG oxygenases essential for epigenetic reprogramming in macrophages through histone demethylation. Remarkably, the increase in the succinate/2-OG ratio inhibits PHD and JMJD function activating HIF-1α and sustaining the glycolytic switch and proinflammatory phenotype (110). In this way the HIF-ROS axis represents a central functional cycle of mutual support where ROS production is a mediator of hypoxia adaptation, by translating oxygen limitation into transcriptional regulation for a metabolic reprogramming.

More recently, it has been described that SARS-CoV2 infection triggers mtROS production in monocytes, and this induces HIF-1α stabilization and consequently promotes glycolytic reprogramming, thereby enhancing the viral replication (111). SARS-CoV2 infection induced downregulation of several proteins of the ETC, such as NDUFV1 (complex I), SDHA (complex II), UQCRC2 (complex III), limiting oxygen consumption rate (111). Defects of the mitochondrial respiratory chain have been related to increased mtROS levels (45), although the precise mechanisms linking infection and mtROS production are still to be defined.

### 3.3 NF-κB Pathway

NF-κB is a transcription factor that plays a crucial role in inflammatory and immune responses (112). It displays a plethora of modulatory mechanisms due to the different DNA binding affinities of their homo- and hetero-dimeric complexes emanating from the five monomers (RelA/p65, RelB, cRel, NF-κB1 p50, and NF-κB2 p52). This heterogeneity is further increased by interactions of the NF-κB dimers with other transcription factors (112). The heterodimeric complex p50/p65 is one of the better characterized during the inflammatory response (113). NF-κB in the cytosol is inactivated by binding to the regulatory protein IkBα (nuclear factor of kappa light polypeptide gene enhancer in B-cells inhibitor, alpha). Inflammatory stimuli drive the phosphorylation of the IKK complex, consisting of two catalytic (IKKα and IKKβ) and one regulatory subunit (NEMO or IKKϒ), the latter acting as scaffold protein for IKKβ activity. The activated complex phosphorylates IκBα, marking the protein for proteasome-linked degradation (114). NF-κB is then free to translocate to the nucleus and start the transcription of several genes, including inflammatory and antiapoptotic genes. The mechanisms linking ROS formation to NF-κB activation are multiple. In macrophages, mtROS are known to mediate IKK complex activation by forming a disulfide bridge between Cys54 and Cys347 on NEMO, which is crucial for IKK complex activation (115). Under proinflammatory stimulus, the IKK complex can be activated by ROS produced by the GTP-binding protein Rac1 (116), leading to signal transduction pathways that contribute to TNF-α secretion. In line with this finding, ROS effects can be suppressed by SOD, reducing the pro-inflammatory immune responses by blocking the p38-MAPK/NF-κB signaling activation (117).

NF-κB is also involved for different transduction outcomes related with anti-inflammatory response. This is particularly evident in the context of the tumor microenvironment, in which ROS formation activates NF-κB signaling, which binds the *Pdl1* promoter in a transcriptional specific manner, leading to PD-L1 expression and release of immunosuppressive chemokines. Indeed, the described ROS-mediated NF-κB activation does not induce expression of the classical NF-κB target IL-6 (118). Similarly, in the context of colitis, mtROS lead to induction of NF-κB signaling responsible of a protective effect associated to the recruitment and polarization of intestinal macrophages to the M2 anti-inflammatory phenotype (119).

Notably, NF-κB is involved also in hypoxic conditions, and evidence of a crosstalk between NF-κB and HIF-1α is growing. PHD inhibition regulates the activity of IKKβ, inducing the nuclear translocation of NF-κB (120). Conversely, hypoxia-mediated NF-κB induction controls HIF-1α activity in macrophages, enhancing the production of pro-inflammatory cytokines and chemokines to sustain the host defense response (121). The link between NF-κB and HIF-1α is further underlined by the finding that HIF-1α promoter contains a NF-κB binding site that can trigger HIF-1α upregulation under conditions of NADPH oxidase-mediated ROS formation (122).

The crosstalk between HIF and NF-κB pathways is extensive, intensive (123) and bi-directional (124). In fact, they are reciprocally regulated (124). An additional level of functional crosstalk between HIF and NF-κB includes common activating stimuli, shared regulators and target genes (123). The overlap of common regulators between HIF and NF-κB consequently finds functional involvement of HIF in processes in which NF-κB is involved, such as infection and inflammation. Many important genes are regulated by HIF and NF-κB (123), including cytokines and chemokines, such as TNF-α, IL- 1β and IL-8. In addition, cell death related proteins, such as Noxa and BNIP3, and other important cellular proteins such as PKM2, Tert, Cyclin D1, and Cox-2 are also shared HIF and NF-κB targets. However, it is not known whether these genes are targeted by these transcription factors at the same time or independently of each other (123).

Moreover, HIF and NF-κB synergistically respond against pathogens. In fact, it has been demonstrated that macrophages, infected by Gram-negative and Gram-positive bacteria, are characterized by a defective HIF-1α expression following the ablation of IKKβ, essential regulator of NF-κB activity (121, 125).

### 3.4 Jak/STAT Pathway

Signal transducer and activator of transcription (STAT) proteins are a family of transcription factors that are essential for the cellular response to cytokines and growth factors. STATs are latent in the cytoplasm under resting conditions. When extracellular stimuli, such as cytokines, bind to specific cell-surface receptors, they activate the tyrosine kinases Jak (‘Janus kinase’), that phosphorylates STAT proteins, thereby allowing translocation to the nucleus to drive transcription of several chemokines and cytokines. The JAK-STAT signaling pathway is fundamental for the immune system (126–129). So far, seven members of the family (STAT1-4, STAT5A, STAT5B, and STAT6) have been identified. STATs are fundamental in the inflammatory/anti-inflammatory response, like the antiviral response through interferon production, and in wound healing (130, 131). The JAK-STAT pathway plays an essential role in macrophage polarization: STAT1 shifts macrophages towards a pro-inflammatory M1 profile activated by interferon gamma (IFN-γ), whereas STAT6 is associated to an M2 anti-inflammatory profile through IL-4. The role of ROS during STAT activation in macrophages is still under scrutiny. A positive feedback in the ROS-p38MAPK-STAT1 axis has been described, as STAT1^-/-^ mice showed impairment in p38MAPK activation in a ROS-dependent manner (132). As a further suggestion of a cooperative role between ROS and STAT1 activation, in NOX-deficient diabetic mice the reduced ROS levels were found to impair STAT1 activation, and to increase STAT6 activation, thereby promoting a M2 signature during diabetes progression (133). On the other hand, the H_2_O_2_ produced by Cu-Zn SOD activity in alveolar murine macrophages was found to activate STAT6 by redox regulation of a critical cysteine during the polarization toward M2-like macrophages (134). Taken together, these findings suggest that further studies will be warranted to understand the tight crosstalk between ROS and STAT, considering the sources and the kind of ROS which are involved.

### 3.5 STING Pathway

The Stimulator of Interferon Genes (STING) pathway senses cytosolic double-strand (ds) DNA, that is a sign of microbial infection, cell injury or nuclear DNA damage (135). Cytosolic dsDNA triggers the activation of the cyclic-GMP-AMP-synthase (cGAS), leading to endogenous generation of cyclic GMP-AMP (cGAMP), a unique second messenger. cGAMP binds to and activates the endoplasmic reticulum transmembrane receptor STING, finally resulting in the production and release of type I interferons (IFN), which are potent anti-viral and anti-cancer cytokines (136). On the other hand, the hyperactivity of STING pathway has been implicated in several debilitating autoimmune syndromes (137–139) (i.e. systemic lupus erythematosus) and in acute and chronic inflammation (140).

STING forms a domain-swapped homodimer in the absence of ligands, whereas, upon cGAMP binding, it undergoes extensive conformational rearrangements, leading to oligomerization (141). Recently, a structural analysis by Ergun et al. demonstrated that STING polymerization is necessary for its activation through the formation of intermolecular disulfide bonds *via* Cys148 (142). Moreover, the increase of mtROS led to cGAS-STING induction of type I IFN (143, 144). Controversially, during herpesvirus infection ROS were found to dampen the type I IFN production in a STING-dependent manner (145). To try to reconcile these findings, the authors speculated that the differences could be ascribed to the amount of ROS, as cysteines can be susceptible to different post-translation modifications, which can either activate or inhibit protein function. More in detail, high ROS levels could oxidize STING thus preventing its polymerization and interferon production, whereas lower levels can promote its assembly. Thus, further studies will be warranted to elucidate how the post-translational modifications of STING by redox regulation affect the innate immune responses against DNA viruses, especially to identify novel immunotherapy targeting IFN production.

### 3.6 Thioredoxins and ASK1

Thioredoxins (TRXs) are small proteins that represent a key protection system against oxidative stress through their disulfide reductase activity. TRXs contain two redox-active cysteines in a Cys-X-X-Cys motif, that can be reversibly oxidized to keep intracellular redox balance (146). Besides the reducing activity, TRXs are also important components of redox signaling pathways. Indeed, TRXs control the activity of several proteins by direct physical interaction (146). For instance, TRX binds the apoptosis signal-regulating kinase 1 (ASK1), thus forming an inactive TRX-ASK1 complex. An increase of intracellular ROS induces disulfide bond formation in TRX. Such a conformational change allows ASK1 release, which activates p38MAPK and NF-κB pathways, and triggers a cell death program (147–149). This axis is supported in macrophages, as LPS stimulation, in a Myd88- dependent manner, induces the production of H_2_O_2_ to activate NF-κB (148). Remarkably, during tuberculosis infection (150) the recognition of tuberculin protein by TLR2 triggered an increase in ROS production that enhanced a positive feedback burst of ROS by TRX-ASK1-p38 activation (150). Another mechanism, described in microglia, indicates that ASK1 activation occurs by sensing extracellular ATP through the P2X7 receptor, which triggers ROS generation leading to TRX-ASK1 release (151). Furthermore, TRX binds to the thioredoxin-interacting protein (TXNIP). TXNIP activation appears to be essential for NLRP3 assembling by different inflammasome agonists, such as monosodium urate crystals (MSU), ATP or high concentration of glucose (152). These stimuli increase ROS production that allows TRX cysteine oxidation, inducing the breakdown of TRX-TXNIP interaction, and allowing TXNIP to bind NLRP3 to stabilize the inflammasome assembly (152). Moreover, endoplasmic reticulum stress induces ROS-mediated IRE1α activation, that increases the TXNIP mRNA stability, enhancing the NLRP3 activation in sterile inflammation (153).

## 4 Conclusions and Perspectives

The studies summarized in this review highlight the crucial and versatile functions of cytosolic and mitochondrial ROS in macrophages. The signaling and damaging properties of ROS can drive the inflammatory response, as well as the diseases resulting from chronic or overwhelming inflammation. The redox balance depends on many parameters, including the levels and the compartmentalization of ROS, their specific sources and subspecies and the specificity/selectivity for their targets.

The great impact of oxidative stress in many diseases explains the enormous number of studies and clinical trials targeting ROS for therapeutic purposes. However, non-selective antioxidants at high doses did not prove effective in either preventing or treating disease processes. This is probably to be ascribed to the disruption of crucial intracellular redox signaling. Even worse, clinical trials have showed harmful effects of antioxidants (154). Indeed, the physiological relevance of H_2_O_2_ signaling was still unclear when most of these trials were performed (6, 155). It is now widely accepted that ROS are part of a signaling network with different sources and targets. Their different subcellular compartmentalization and expression suggest the presence of multiple hot spots of ROS with different roles, rather than a homogenous intracellular redox level. The expanding knowledge about the pleiotropy of ROS signaling requires that therapeutic interventions use strategies aimed at addressing the specific disease-relevant mechanisms without disrupting other crucial signaling pathways.

With this respect the development of novel therapeutic approaches targeting ROS will require to identify and dampen the main sources of deleterious ROS relevant for a specific disease, without altering the vital physiological sources of ROS and their downstream signaling pathways. The clinical status of these mechanism-based redox therapies is summarized in (154). An example is the inhibition of specific NOX isoforms or the use of Nrf2 agonists enhancing the expression of endogenous antioxidant enzymes at their physiological sites. Our studies support the relevance of targeting the mitochondrial enzyme MAO to counteract inflammatory diseases for several reasons. Indeed, MAO inhibitors, for which the mechanism of action, pharmacodynamics and pharmacokinetics are well established (they are approved drugs from Parkinson’s disease), block the formation of a specific subset of mitochondrial ROS relevant in pathological conditions, thus preventing mitochondrial dysfunction. More research targeting specific ROS sources and defined mechanisms are strongly awaited.

## Author Contributions

MC, RS-R, and AC: conceptualization, writing original draft, reviewing, and editing. AV: supervision, reviewing. IS and MF: supervision, literature searching, reviewing, and editing. FV: supervision, reviewing, and editing the figures. All authors contributed to the article and approved the submitted version.

## Funding

The study was supported by ERC-2019-PoC n. 899770 to AV and IRP-PENTA Grant 18/07-1 to MC.

## Conflict of Interest

The authors declare that the research was conducted in the absence of any commercial or financial relationships that could be construed as a potential conflict of interest.

## Publisher’s Note

All claims expressed in this article are solely those of the authors and do not necessarily represent those of their affiliated organizations, or those of the publisher, the editors and the reviewers. Any product that may be evaluated in this article, or claim that may be made by its manufacturer, is not guaranteed or endorsed by the publisher.
